# Monocyte–red blood cell crosstalk supports clearance and heme metabolism in sickle cell anemia

**DOI:** 10.3389/fimmu.2025.1699306

**Published:** 2025-12-05

**Authors:** Marina Dorigatti Borges, Matheus Ajeje de Souza, Izabela Felice Paes, Daniela Pinheiro Leonardo, Dulcinéia Martins Albuquerque, Carolina Lanaro, Sara Teresinha Ollala Saad, Renata Sesti-Costa, Fernando Ferreira Costa

**Affiliations:** Laboratory of Hemoglobin and Genome - Hematology and Hemotherapy Center, University of Campinas (UNICAMP), Campinas, SP, Brazil

**Keywords:** erythroblastic island, macrophages, hemolytic anemia, sickle cell disease, iron metabolism

## Abstract

**Background:**

Monocytes can interact with erythroid cells and contribute to macrophage pools under anemic stress, thereby supporting the erythropoietic niche. In sickle cell anemia (SCA), extensive hemolysis and ineffective erythropoiesis may expose circulating monocytes to abnormal red blood cells (RBCs), potentially altering their phenotype and function to accommodate the higher erythropoietic response and iron demand.

**Methods and results:**

We characterized circulating monocytes from SCA patients at steady state using flow cytometry and *in vitro* phagocytosis assays. Intracellular RBC material was detected in all circulating monocyte subsets from SCA patients, indicating active erythrophagocytosis. Mechanistically, SCA monocytes displayed upregulated VCAM-1 and reduced SIRP-α expression, favoring RBC binding and internalization even when CD47 expression on RBCs was preserved. Following RBC engulfment, monocytes upregulated heme oxygenase-1 (HO-1) and ferroportin (FPN), consistent with enhanced heme degradation and iron export, and expressed higher levels of CD206, suggesting a regulatory phenotype. *In vitro* assays confirmed that both sickle RBCs and SCA monocytes synergistically promoted erythrophagocytosis.

**Conclusion:**

Circulating monocytes from SCA patients undergo phenotypic and functional reprogramming upon interaction with sickled RBCs, acquiring features reminiscent of erythroblastic island macrophages. These findings highlight a previously underappreciated role for monocytes in RBC clearance, heme metabolism, and iron recycling in SCA, with potential implications for inflammation and disease progression.

## 1 Introduction

Erythroid cells remain closely associated with macrophages throughout several stages of their development, particularly because erythropoiesis occurs within specialized niches known as erythroblastic islands (EBI) (1, 2). These niches consist of erythroid cells at various stages of maturation organized around a central macrophage. The functions of this central macrophage include supplying iron for hemoglobin synthesis, promoting proliferation and survival of erythroid precursors, and engulfing extruded nuclei during terminal differentiation. In addition, macrophages in the liver and splenic red pulp are responsible for the phagocytosis of damaged or senescent red blood cells (RBC) (3–9).

Monocytes also have the capacity to differentiate and contribute to the macrophage pool, particularly under anemic conditions (10), and appear to establish close interaction with erythroid cells even prior to their differentiation into macrophages. For instance, monocytes can induce erythropoiesis, repress gamma globin synthesis, and contribute to the clearance of damaged or senescent RBCs from circulation (11–13). One of the main functions of tissue-resident macrophages is the uptake of heme from the complex haptoglobin-hemoglobin via the receptor CD163 (14). Circulating monocytes express CD163 at much lower levels than resident macrophages and are therefore not the primary cells responsible for hemoglobin clearance. Nevertheless, in inflammatory contexts, CD163 can be upregulated on monocytes, conferring them a limited scavenging capacity (15–18).

Sickle cell anemia (SCA) is a hereditary disorder caused by a point mutation in the sixth codon of the β-globin gene, resulting in the substitution of valine for glutamic acid. Consequently, hemoglobin A (HbA) is replaced with hemoglobin S (HbS), which is prone to polymerization under low oxygen conditions. HbS polymerization alters the morphology of erythroid cells, converting them from flexible, biconcave discs into rigid, sickle-shaped cells, thereby reducing deformability and causing membrane damage (19–21).

In SCA, approximately 40% of erythroid cells die between the polychromatic and orthochromatic erythroblast stages, overloading EBI macrophages due to the increased burden of apoptotic cells (22, 23). Kupffer cells are likewise affected, as the heightened erythrophagocytosis triggered by membrane damage in sickled RBCs leads to hyperplasia and hepatomegaly (9). Monocytes, in turn, have been reported to participate in the clearance of RBCs adherent to the endothelium of SCA patients at steady-state and during vaso-occlusive crises. They also display phenotypic adaptations to meet the increased iron demand and are activated by hemolysis and the consequent erythropoietic response (13, 24, 25). The specific role of monocytes in SCA and the influence of disease features in their function has been investigated through single-cell and functional studies in recent years (26–29).

With this in mind, we sought to characterize the phenotypic and functional properties of circulating monocytes in SCA patients, with particular emphasis on their ability to interact with and engulf RBCs, and to evaluate the impact of this interaction on iron metabolism and monocyte activation status, suggested by the expression of key markers. Our data demonstrated that circulating monocytes from these patients exhibit phenotypic and functional alterations consistent with active interaction with erythroid cells. These findings support the hypothesis that, in SCA, circulating monocytes undergo functional reprogramming upon contact with abnormal erythrocytes, acquiring features reminiscent of macrophages within EBIs.

## 2 Materials and methods

### 2.1 Sample collection

Patients with SCA were recruited at Blood Center of the University of Campinas (UNICAMP). The exclusion criteria were transfusions, hospitalizations, and pain crisis for at least 3 months prior to blood collection. All patients were prescribed hydroxyurea; six of them were unable to access the medication in the three months preceding sample collection. The control group consisted of healthy individuals (HC), blood donors in the same institution, matched by age and sex with the patients. Ethical approval was obtained from the UNICAMP Human Research Ethics Committee (protocol number CAAE: 88768318.6.0000.5404), and all participants provided written informed consent. Demographic characteristics are detailed in **Table 1**.

**Table 1:** Main demographic, hematological, and laboratorial characteristic of sickle cell anemia patients’ population.

	Median (min-max)
Age (n=39)	43 (20–60)
RBC (3.9-6x10^6^/ul) (n=39)	2.28 (1.53-3.49)
Platelets (130-400x10^3^/ul) (n=39)	334 (122-720)
WBC (3.7-11.1X10^3^/ul) (n=39)	5.37 (3.55-11.95)
MHC (27.3-32.6pg) (n=39)	33.7 (27.5-44.9)
MCV (82-98fl) (n=39)	115.4 (84.4-139.9)
HGB (11.8-16.7g/dL) (n=39)	8.3 (6-11)
HCT (36-50%) (n=39)	26.2 (17.5-32.6)
Monocytes (0.2-0.92x10^3^/ul) (n=39)	0.33 (0.12-1.27)
Serum iron (70-180ug/dL) (n=32)	116.5 (38-208)
Ferritin (m:30-400; f:13-150ng/Dl) (n=32)	593 (25.1-3039)
TIBC (255-450ug/dL) (n=32)	272.5 (154-411)
Transferrin saturation (20-45%) (n=32)	40.5 (11-96)
Indirect bilirubin (0.10/1mg/dL) (n=22)	1.52 (0.81-6.24)
Direct bilirubin (0.3-1.2mg/dL) (n=22)	1.78 (0.44-6.94)
HbF (%) (n=33)	16.1 (1.6-30.5)
HbS (5) (n=33)	77.1 (53.9-91.4)
	**n**
Sex (male/female) (n=39)	17/22
Use of hydroxyurea (n=39)	33

RBC, red blood cells; WBC, white blood cells; MCH, mean corpuscular hemoglobin; MCV, mean corpuscular volume; HGB, hemoglobin; HCT, hematocrit; TIBC, total iron binding capacity; HbF, fetal hemoglobin; HbS, hemoglobin S; n, sample size.

### 2.2 Peripheral blood mononuclear cell isolation

Peripheral blood mononuclear cells (PBMCs) were isolated from 20mL of whole blood by density gradient separation using Ficoll-Hypaque (Cytiva). The blood was diluted with sterile phosphate-buffered saline (PBS), carefully layered on top of Ficoll-Hypaque, and centrifuged at 1500 rpm for 30 minutes at room temperature (without acceleration or brake). The PBMC layer was collected and washed twice with PBS. The remaining RBCs were lysed with chilled lysis buffer (0.155 M NH_4_Cl and 0.0010 M KHCO_3_) at 4 °C for 15 minutes. Following an additional wash, the cells were resuspended and quantified.

### 2.3 Monocyte analyses

The phenotypic characterization of circulating monocytes was performed via flow cytometry (FC) using the Cytoflex equipment (Beckman Coulter). Cell populations were initially gated based on forward and side scatter (FSC × SSC) parameters, followed by the exclusion of doublets using SSC-A × SSC-H. Based on CD14 and CD16 expressions, monocytes were categorized into three subsets: classical (C-MC, CD14^++^CD16^-^), intermediate (I-MC, CD14^++^CD16^+^), and non-classical (NC-MC, CD14^+^CD16^+^). The expression of target molecules was assessed using fluorescent antibodies. Antibody details are provided in **Supplementary Table 1**. The mean fluorescence intensity (MFI) of the different markers were accessed in each monocyte subset.

The RBCs phagocytosis was evaluated following the protocol described by Liu et al. (25). Monocytes were stained with anti-CD14 and anti-CD16 antibodies, fixed, and permeabilized using the Cytofix/Cytoperm kit (BD Biosciences, 554714) according to the manufacturer instructions before intracellular staining with an anti-glycophorin A antibody. The data was analyzed using FlowJo software (BD Biosciences).

### 2.4 *In vitro* phagocytosis assay

The methodology described by Haschka et al. (13) was adapted as follows: monocytes were isolated from PMBCs using anti-CD14 magnetic beads (Miltenyi Biotec) according to the manufacturer’s instructions and allowed to adhere overnight. RBCs from patients or HCs were isolated from peripheral blood, labeled with the membrane dye PKH26 (Sigma), and co-cultured with total monocytes for 2 hours to allow phagocytosis (5×10^6^ cells RBCs and 5×10^5^ monocytes per well). Subsequently, cells were collected, and remaining RBCs were lysed with chilled lysis buffer containing NH_4_Cl 0.155M and KHCO_3_ 0.0010M at 4°C for 15 minutes, to clear the adherent cells. Monocyte were gated in the singlet population and PKH26 fluorescence was evaluated by MFI and percentage. Ferroportin (FPN) expression on the cell membrane was assessed using an anti-FPN primary antibody followed by incubation with a fluorescent secondary antibody. Intracellular expression of heme oxygenase-1 (HO-1) was evaluated by staining with an anti-HO-1 antibody after fixation and permeabilization using the Cytofix/Cytoperm kit.

### 2.5 Statistical analyses

Statistical analyses were performed using Prism 5.1 software (GraphPad). P-values ≤0.05 were considered statistically significant. Results are expressed as mean ± standard deviation. Statistical significance was determined using a two-tailed paired t-test or the Mann-Whitney test, as indicated in the figure legends.

## 3 Results

### 3.1 Circulating monocytes contribute to the phagocytosis of RBCs in SCA patients

**Figure 1:**
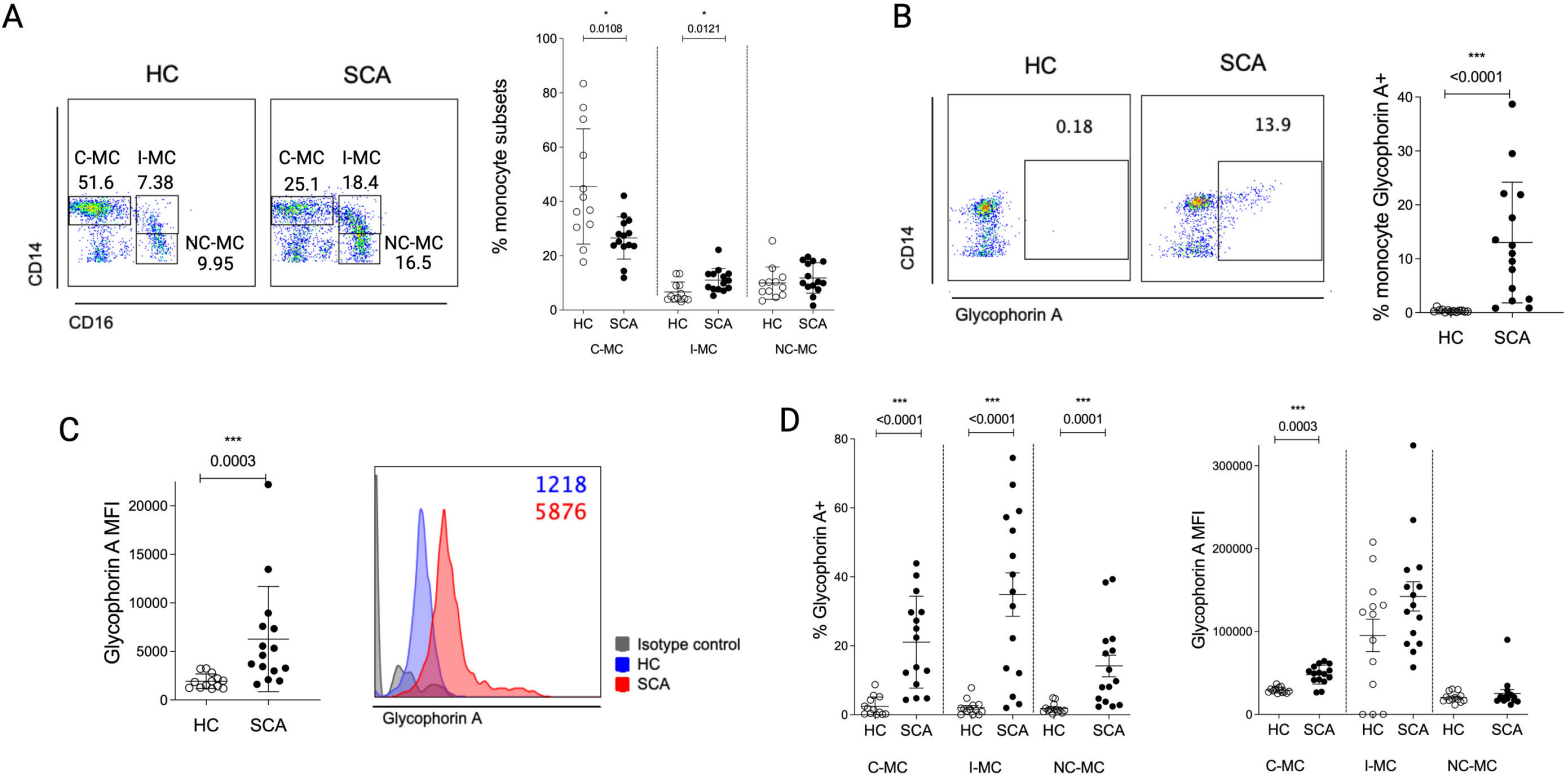
Phagocytosis of erythroid cells by circulating monocytes in sickle cell anemia patients (SCA). **(A)** Flow cytometry analysis of circulating monocytes (CD14^+^) from PBMC of healthy controls (HC) and SCA patients subdivided into the main subsets: C-MC: classical monocytes; I-MC: intermediate monocytes; NC-MC: non-classical monocytes. Percentage **(B)** mean fluorescence intensity (MFI) **(C)** of glycophorin A in total circulating monocytes. **(D)** Percentage and MFI of glycophorin A in each monocyte subset. HC, n = 13; SCA, n = 15.

Flow cytometry analysis of circulating monocyte subsets in the present cohort showed an increased proportion of intermediate monocytes (CD14^+^CD16^+^) and reduced classical monocytes (CD14^++^CD16^-^) in SCA patients compared to healthy controls (HC) (**Figure 1A**). It was consistent with a previous study that demonstrated a similar shift in monocyte subsets among patients under hydroxyurea (HU) therapy (30). Since monocytes from SCA patients have previously been reported to participate in RBCs phagocytosis (20), we evaluated their involvement in this process in our cohort by assessing the intracellular presence of glycophorin A, a membrane glycoprotein of erythroid cells, within circulating monocytes. While monocytes from HC displayed minimal basal internalization of RBCs, those from SCA patients exhibited markedly higher percentages and mean fluorescence intensity (MFI) of intracellular glycophorin A (**Figures 1B, C**). These findings corroborate previous reports, reinforcing the role of these monocytes in the clearance of erythroid cells from circulation. When analyzing the proportion of glycophorin A^+^ cells across monocyte subsets, all three subsets were involved, with I-MC displaying the highest percentage of RBCs uptake (**Figure 1D**), and C-MC from SCA patients showing significantly increased glycophorin A MFI compared to HC (**Figure 1D**).

### 3.2 Mechanisms involved in erythrophagocytosis by circulating monocytes from SCA patients

**Figure 2:**
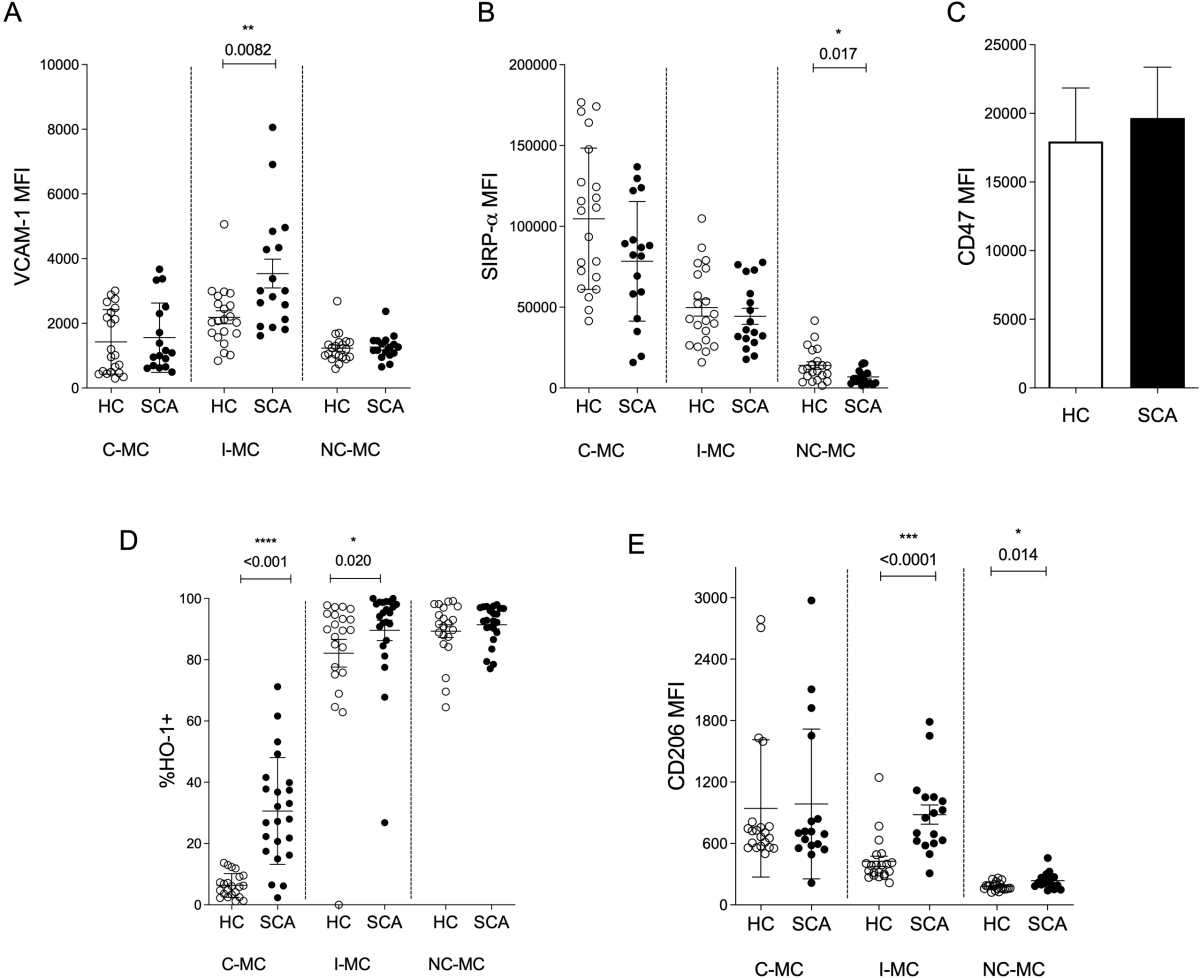
Phenotypic characterization of circulating monocytes in SCA patients. Mean fluorescence intensity (MFI) of VCAM-1 **(A)**, SIRP-α **(B)** and CD206 **(E)** on healthy controls (HC) and SCA monocyte subsets: C-MC: classical monocytes; I-MC: intermediate monocytes; NC-MC: non-classical monocytes. **(C)** MFI of CD47 in RBCs. **(D)** Percentage of HO-1^+^ monocytes. HC, n = 13; SCA, n = 15.

Erythrophagocytosis by macrophages involves cell attachment mediated by adhesion molecules and the interaction between SIRP-α and CD47. SIRP-α, expressed on macrophages and monocytes, recognize CD47 on healthy RBCs. The loss of CD47 on senescent or damaged RBCs, or the downregulation of SIRP-α on phagocytes, results in the clearance of RBCs from circulation (31). To explore potential mechanisms influencing the engulfment of erythroid cells by circulating monocytes in SCA patients, we analyzed the expression of VCAM-1 and SIRP-α on monocytes, as well as CD47 on RBCs. VCAM-1, a major adhesion molecule mediating interactions between erythroid cells and monocytes, was upregulated on I-MC from SCA patients compared to HC (**Figure 2A**), suggesting an enhanced ability of these monocytes to interact with and adhere to circulating RBCs. Furthermore, all monocyte subsets from SCA patients exhibited reduced SIRP-α expression compared to their HC counterparts, although the reduction was statistically significant only in NC-MC (**Figure 2B**). These correlative findings suggest an impaired capacity to recognize CD47 on RBCs, potentially promoting their phagocytosis even when CD47 is normally expressed on sickle RBCs (**Figure 2C**).

In line with the increased uptake of RBCs, all monocyte subsets from SCA patients displayed upregulated expression of heme oxygenase-1 (HO-1), with statistical significance for C-MC and I-MC and a similar trend for NC-MC (**Figure 2D**), indicating an enhanced capacity to metabolize heme derived from RBC engulfment. Moreover, both I-MC and NC-MC from SCA patients exhibited higher CD206 expression compared to HC, suggesting a shift toward a more regulatory phenotype (**Figure 2E**).

### 3.3 Influence of monocytes and RBCs on phagocytosis and iron metabolism in SCA

**Figure 3:**
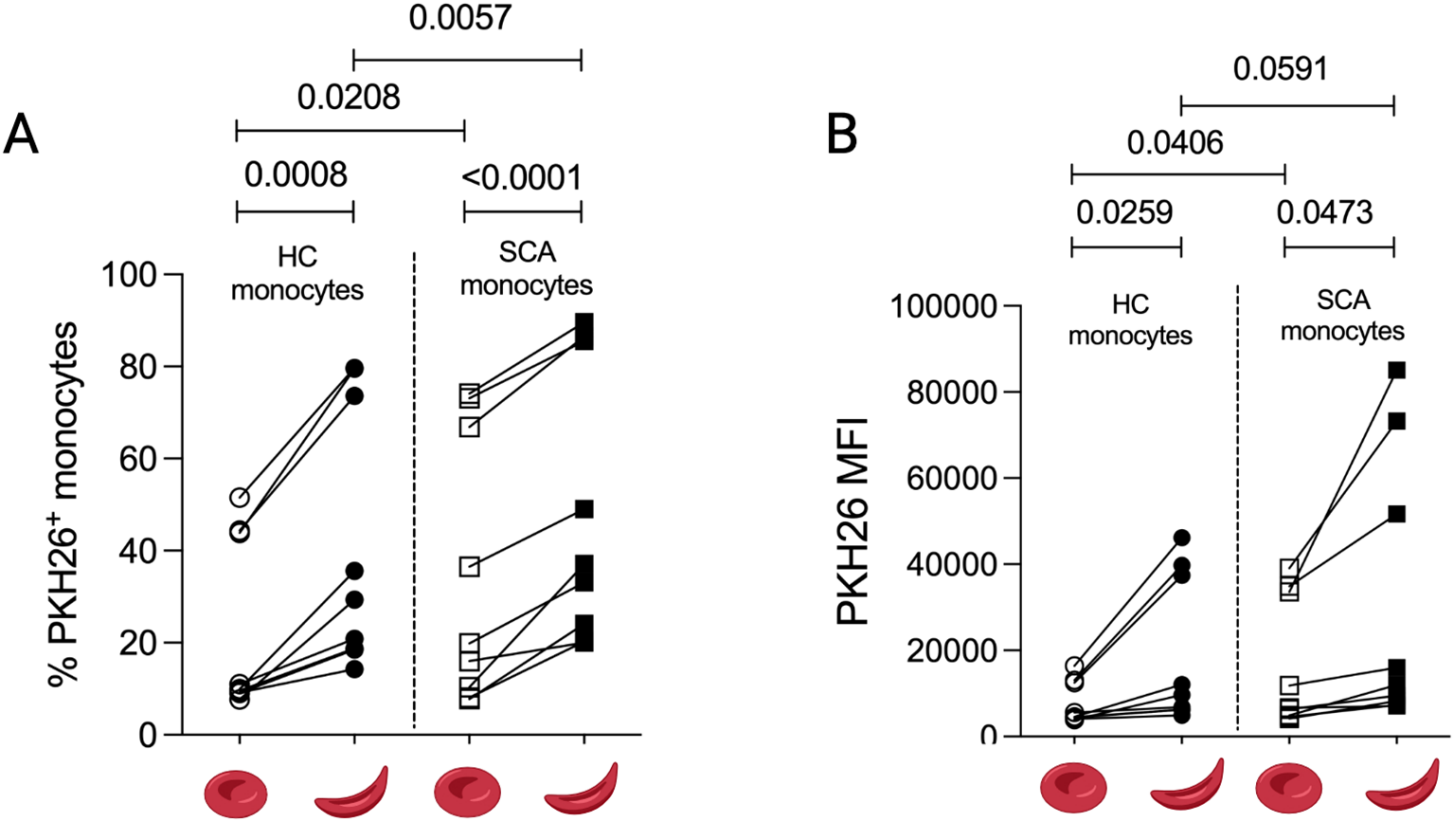
Erythrophagocytosis assay of circulating monocytes. RBCs from healthy controls and SCA patients were PKH26-labeled and incubated with allogeneic monocytes either from HC (HC monocytes) or from SCA patients (SCA monocytes) for 2 h. The graphs show percentage **(A)** and mean fluorescence intensity (MFI) **(B)** of PKH26 in total monocytes. HC, n = 6; SCA, n = 6.

To investigate the interaction between monocytes and RBCs, we performed an *in vitro* phagocytosis assay by incubating monocytes separated by CD14-selective magnetic beads and PKH26-labeled RBCs from either HC or SCA patients, followed by quantification of PKH26 signal within monocytes after lysis of adherent RBCs. After co-culture, monocytes from both groups interacted significantly more with SCA RBCs than with HC RBCs, as evidenced by higher percentage of PKH26^+^ cells and increased PKH26 MFI (**Figures 3A, B**), highlighting the role of sickle RBCs in promoting their engulfment. In addition, monocytes from SCA patients interacted to a greater number of RBCs compared to HC monocytes, both when incubated with HC RBCs and with SCA RBCs (**Figures 3A, B**). These findings indicate that both RBCs and monocytes from SCA patients contribute to enhancing this interaction, thereby facilitating RBC clearance, as previously described.

**Figure 4:**
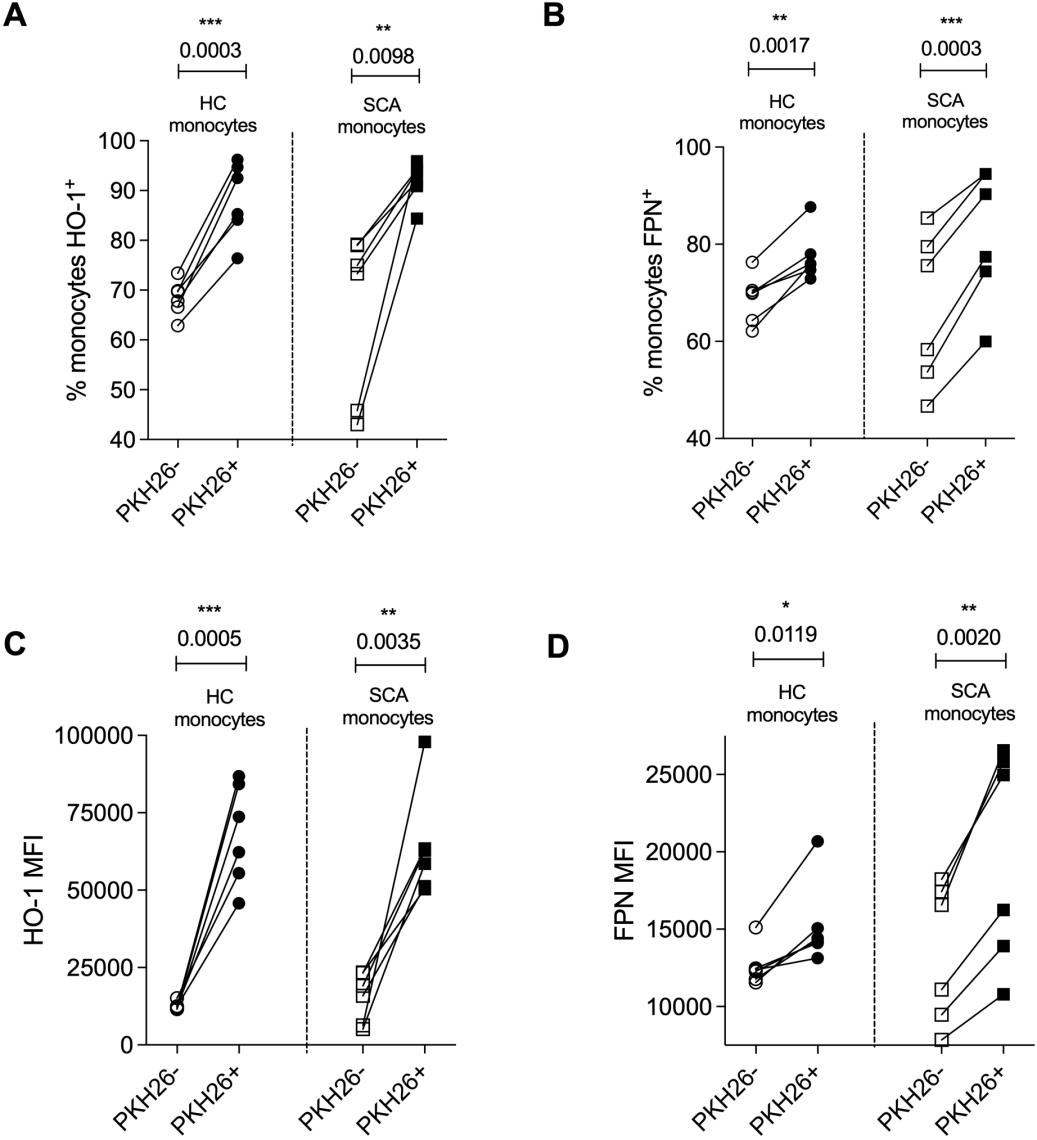
Expression of heme oxygenase-1 (HO-1) and ferroportin (FPN) after engulfment of sickle cell anemia patients red blood cells. Percentage of HO-1^+^**(A)** and ferroportin (FPN^+^) **(B)** among PKH26^+^ and PKH26^-^ monocytes from healthy controls (HC monocytes) and SCA patients (SCA monocytes); Mean of fluorescence intensity (MFI) of HO-1 **(C)** and FPN **(D)** among PKH26^+^ and PKH26^-^ monocytes. HC, n = 6; SCA, n = 6.

To investigate the impact of RBCs engulfment by circulating monocytes, we assessed the expression of HO-1 and ferroportin (FPN), two key proteins involved in heme and iron metabolism. When comparing monocytes that had internalized SCA RBCs (PKH26^+^) with those that had not participated in the phagocytosis/interaction process (PKH26^-^), we found that HO-1 and FPN expression were increased in both HC and SCA monocytes following contact with RBCs (**Figures 4A-D**). These findings suggest that circulating monocytes internalize RBCs, thereby promoting heme degradation and iron export, possibly as a compensatory mechanism for the increase of intracellular load.

## 4 Discussion

In the present study, we characterized circulating monocytes from patients with SCA and identified key phenotypic and functional alterations associated with their interaction with erythroid cells. Our findings highlight a dynamic interplay between circulating monocytes and sickled RBCs, potentially contributing to systemic changes in both inflammation and iron homeostasis in SCA. While monocytes from HC do not engulf RBCs normally in the circulation of these individuals, our *ex vivo* analyses revealed the presence of RBCs material in monocytes from SCA patients, consistent with RBC binding and internalization, as previously described (20).

VCAM-1 expressed by macrophages plays a key role in adhesion to RBCs through binding to α4β1 integrin (29). We observed that circulating monocytes from SCA patients expressed higher levels of VCAM-1, suggesting one mechanism by which monocytes interact with RBCs in this context. Moreover, SIRP-α, an important inhibitor of erythrophagocytosis, was reduced in monocytes, mainly in NC-MC, from SCA patients. Given that all monocyte subsets interact with or contained intracellular remnants of erythroid cells, and that no correlation was seen between SIRP-α and glycophorin A MFI (data not shown), our findings suggest that SIRP-α, although relevant, is neither the sole nor the dominant regulator of this process. Nevertheless, reduced SIRP-α expression may imply that these monocytes are more actively engaged in RBC clearance, due to impaired recognition of CD47. Supporting this view, Pan and colleagues (48) demonstrated increased phagocytic activity in monocytes and neutrophils from Sirp-α knock-out mice (22), while Londino et al. showed that loss of SIRP-α enhances inflammatory signaling (32). Indeed, the SIRP-α-CD47 axis seems to be particularly critical under inflammatory conditions. Bian and colleagues reported that blocking this interaction in mice under physiological conditions had no impact on macrophage phagocytosis; however, during inflammation it resulted in severe anemia (33), emphasizing how inflammation modulates this pathway. In our cohort of hydroxyurea (HU)-treated patients, sickle RBCs exhibited CD47 expression comparable to normal RBCs, indicating that CD47 loss does not account for RBCs interaction. However, since CD47 levels increase with HU therapy (34), reduced CD47 expression in untreated patients may contribute to the enhanced phagocytosis of sickle RBCs by circulating monocytes. Additional functional assays targeting SIRP-α/CD47 and VCAM-1/α4β1 integrin would further clarify and quantify the involvement of SIRP-α and VCAM-1 in the process.

Our data demonstrated that monocytes from SCA patients upregulated intracellular HO-1 expression, suggesting a response to heme from phagocytosed RBCs or RBC microvesicles (35–38). This finding further supports the internalization of part of sickled RBCs or RBCs microvesicles by circulating monocytes, consistent with previous reports showing increased of HO-1 expression following phagocytosis (25). Macrophages with enhanced phagocytic and scavenging activity, such as the central macrophages within EBIs, have been shown to express high levels of CD206, a marker of alternatively activated cells (39–41). In line with this, our cohort revealed that enhanced RBC uptake was associated with increased CD206 expression in SCA monocytes, particularly within I-MC and NC-MC subsets.

Our *in vitro* assays indicates that both monocytes and RBCs from SCA patients contribute to enhanced erythrophagocytosis by circulating monocytes, suggesting that alterations in the surface molecules of both cell types, or structural changes in sickled RBCs, account for their interaction and subsequent internalization. In contrast to previous findings, which indicated that activated endothelial cells are required for sickle RBCs uptake by monocytes *in vitro* (25), our results show that co-culture of monocytes with RBCs alone was sufficient to induce their interaction and RBCs internalization. The upregulation of HO-1 in PKH26^+^ monocytes provides additional evidence of RBC engulfment. However, we acknowledge the limitations of our assays in proving complete internalization of all RBCs, then we cannot exclude the possibility that RBC remnants, such as microvesicles, were internalized, or that some adherent cells were detected. The fact that the patients in this study were receiving HU therapy may also account for these differences, as HU has been reported to alter monocyte phenotype and inflammatory capacity (30). Moreover, the induction of HO-1 and FPN, the only known iron exporter (42, 43), in PKH26^+^ monocytes indicates their capacity to metabolize RBC content, degrading hemoglobin-derived heme and releasing the resulting iron. It is well established that hemoglobin can activate the transcription of both HO-1 and FPN, and that their co-expression facilitates iron recycling by macrophages (44). Notably, the increase in FPN observed as early as 2 hours after the initial contact with RBCs indicates that monocytes are promptly capable of releasing iron. We were not able to detect iron in the supernatant, possibly due to the low number of phagocytosing monocytes in culture. Thus, a definitive proof of iron export and a time-course analysis would be valuable in future studies to further refine these observations.

Although macrophages exert the greatest impact on erythropoiesis and RBCs clearance (45), monocytes also emerge as contributors of erythroid proliferation and survival being found near specific hematopoietic niches in the bone marrow (11). Depletion of CD169^+^ macrophages under homeostatic conditions reduced the number of erythroblasts in the bone marrow, although it did not result in anemia, possibly due to impaired erythrophagocytosis. In contrast, depletion of CD169^+^ macrophages markedly impaired erythropoietic recovery following hemolytic anemia or acute blood loss (3), suggesting that macrophage represent a potential therapeutic target in erythropoietic disorders. Considering the similarities between EBI macrophages and SCA monocytes, we hypothesize that circulating monocytes could enhance erythropoietic turnover induced by anemia, as iron export may support hemoglobin synthesis in newly formed erythroblasts. Moreover, monocytes may assist macrophages in the clearance of RBCs, thereby preventing intravascular hemolysis and the subsequent release of free hemoglobin and heme that trigger chronic inflammation. Depletion of monocytes in animal model of SCA may help to elucidate their contribution to disease pathophysiology.

The main limitation of this study is that most patients were undergoing HU therapy. The remaining patients were prescribed HU but experienced difficulties in obtaining the medication for the three months prior to sample collection. Although these patients did not differ from the overall group, future comparisons between treated and untreated patients will be critical, given the impact of HU on cell phenotypes, monocyte subset distribution, and their direct influence on inflammation and hemolysis (30). HU is also known to modulate the expression of various adhesion molecules on RBCs and may also change SIRP-α expression. Previous studies have demonstrated that HU reduces the expression of α4β1, CD36, VLA-4, and ICAM-4 in erythroid cells from patients with SCA (34, 46, 47). Nevertheless, despite these effects, HU treatment does not fully restore cellular profiles to those of healthy individuals. Future studies including additional patients and independent cohorts will be important to further strengthen and validate the findings presented here.

Our findings offer novel insights into the role of monocytes in RBCs clearance and iron recycling in SCA and establish a foundation for future studies aiming at addressing key questions raised by our results. These include the impact of HU treatment, the potential for monocyte adhesion to erythroid cells at different developmental stages, and the molecular mechanisms that regulate erythrophagocytosis. Collectively, our data highlight circulating monocytes and their interaction to erythroid cells as possible targets for future mechanistic and translational investigations.

## Data Availability

The raw data supporting the conclusions of this article will be made available by the authors, without undue reservation.

## Supplementary material

Supplementary Table 1 List of antibodies used for flow cytometry analysis.
