# Supplementary material for: Monocyte–red blood cell crosstalk supports clearance and heme metabolism in sickle cell anemia
Source: Front Immunol. 2025 Dec 5;16:1699306. doi: 10.3389/fimmu.2025.1699306 (PMC12714641; doi:10.3389/fimmu.2025.1699306)
Supplement: Supplementary Table 1 — List of antibodies used for flow cytometry analysis. [file Table1.docx]

**Supplemental Table 1.** List of antibodies used for flow cytometry analysis.

| **Antibody** | **Clone** | **Function** | **Company** |
| --- | --- | --- | --- |
| CD14-FITC | Tuk4 | Glycoprotein receptor | Life Technologies |
| CD16-PerCP | 3G8 | Type I transmembrane receptor | Life Technologies |
| CD169-647 | 7-239 | Adhesion to erythroid cells | BD |
| CD163-BV421 | GHI/31 | Receptor of hemoglobin-haptoglobin complex, adhesion to erythroid cells | BD |
| Sirp-α-PE | SE5A5 | Phagocytosis signaling | BD |
| VCAM1-BV605 | 51-10C9 | Adhesion to erythroid cells | Biolegend |
| CD206-PE | 19.2 | Mannose receptor, present in regulatory cells | BD |
| Ferroportin- Unconjugated |  | Transmembrane protein that transports iron | LifeSpan Bioscience |
| Goat anti-Rabbit IgG (H+L) Cross-Adsorbed Secondary Antibody, Alexa Fluor™ 633 |  | - | Invitrogen |
| Heme-Oxigenase-1-647 | 23 | Enzyme that breaks down heme | BD |
| HbF-FITC | HBF-1 | Fetal hemoglobin | Life Technologies |
| Glycophorin-PE | CLB-ery-1 | Protein found in the cell membrane of red blood cells | Life Technologies |
